# Attenuation of Bluetongue Virus (BTV) in an *in ovo* Model Is Related to the Changes of Viral Genetic Diversity of Cell-Culture Passaged BTV

**DOI:** 10.3390/v11050481

**Published:** 2019-05-26

**Authors:** Fabian Z. X. Lean, Matthew J. Neave, John R. White, Jean Payne, Teresa Eastwood, Jemma Bergfeld, Antonio Di Rubbo, Vittoria Stevens, Kelly R. Davies, Joanne Devlin, David T. Williams, John Bingham

**Affiliations:** 1 CSIRO Australian Animal Health Laboratory (AAHL), Geelong 3220, Australia; fabian.lean@csiro.au (F.Z.X.L.); matthew.neave@csiro.au (M.J.N.); john.white@csiro.au (J.R.W.); jean.payne@csiro.au (J.P.); teresa.eastwood@csiro.au (T.E.); jemma.bergfeld@csiro.au (J.B.); antonio.dirubbo@csiro.au (A.D.R.); vicky.stevens@csiro.au (V.S.); kelly.davies@csiro.au (K.R.D.); d.williams@csiro.au (D.T.W.); 2 Department of Veterinary Biosciences, Faculty of Veterinary and Agricultural Sciences, University of Melbourne, Parkville 3010, Australia; devlinj@unimelb.edu.au

**Keywords:** *Bluetongue virus*, genetic diversity, attenuation, pathogenicity, immunohistochemistry

## Abstract

The embryonated chicken egg (ECE) is routinely used for the laboratory isolation and adaptation of Bluetongue virus (BTV) in vitro. However, its utility as an alternate animal model has not been fully explored. In this paper, we evaluated the pathogenesis of BTV *in ovo* using a pathogenic isolate of South African BTV serotype 3 (BTV-3) derived from the blood of an infected sheep. Endothelio- and neurotropism of BTV-3 were observed by immunohistochemistry of non-structural protein 1 (NS1), NS3, NS3/3a, and viral protein 7 (VP7) antigens. In comparing the pathogenicity of BTV from infectious sheep blood with cell-culture-passaged BTV, including virus propagated through a *Culicoides*-derived cell line (KC) or ECE, we found virus attenuation in ECE following cell-culture passage. Genomic analysis of the consensus sequences of segments (Seg)-2, -5, -6, -7, -8, -9, and -10 identified several nucleotide and amino-acid mutations among the cell-culture-propagated BTV-3. Deep sequencing analysis revealed changes in BTV-3 genetic diversity in various genome segments, notably a reduction of Seg-7 diversity following passage in cell culture. Using this novel approach to investigate BTV pathogenicity *in ovo*, our findings support the notion that pathogenic BTV becomes attenuated in cell culture and that this change is associated with virus quasispecies evolution.

## 1. Introduction

Bluetongue virus (BTV) is an arthropod-borne (arbo-) virus that is primarily transmitted by *Culicoides* midges. It is a non-enveloped virus, with a 10-segmented double-stranded RNA (dsRNA) genome, belonging to the family *Reoviridae* and genus *Orbivirus*. The infection of susceptible ruminant species can result in viral hemorrhagic disease [1], as with other important human and animal arboviruses, for example, dengue and Rift valley fever [2,3]. Other features of arbovirus infection of vertebrate hosts include the development of in utero congenital neurological diseases, as seen with Zika and Schmallenberg virus infection [4,5]. BTV infection of ruminants was also reported as the cause of neuropathogenic congenital deformities in the fetuses of cattle and sheep [6]. BTV may serve as a model arbovirus to better understand the pathogenesis of viral hemorrhagic disease and congenital neurological diseases arising from arboviral infection.

The pathogenesis of BTV can be evaluated in vivo using natural or laboratory hosts. European-adapted sheep breeds are susceptible host species for BTV infection [7], but ethical considerations and the requirement for a large animal facility with high biocontainment standards limits the use of sheep as an infection model. Alternatively, an interferon (IFN) alpha/beta receptor-deficient mouse model was used for the investigation of BTV pathogenicity [8]. This, however, requires pathogen-free conditions for animal housing [8]. Embryonated chicken eggs (ECEs) are commonly used for BTV isolation as they are the most sensitive laboratory system to demonstrate the presence of infectious virus in clinical specimens and can be easily obtained from reliable sources. This method is recommended by the World Organization for Animal Health (OIE) [9,10]. Although ECEs are routinely used in virology laboratories for BTV isolation, only the pathogenesis of African horse sickness virus (AHSV) was studied using this system [11]. There is currently insufficient knowledge on BTV tropism and pathogenesis in ECE. Previous *in ovo* investigations utilized in situ hybridization to investigate organ tropism of BTV in ECE, however, cellular tropism was not described due the limitations of the techniques employed to detect BTV segment (Seg)-3 [12]. Recently, sensitive BTV immunohistochemistry (IHC) tests allowed improved evaluation and development of a BTV animal infection model [13].

The types of virus inoculum preparations that allow reliable experimental reproduction of BTV infection in animals are not clearly identified, as different results were reported in different studies. Some studies reported that representative clinical bluetongue (BT) disease can be induced with cell-culture-passaged BTV [14,15,16,17,18]. However, numerous investigators advocated against the use of cell-culture-passaged BTV isolates due to attenuated clinical BT disease seen in sheep following experimental infection [19,20,21]. This was recently demonstrated by comparing the pathogenicity of viremic sheep blood and cell-culture-passaged BTV after experimental infection of sheep [7]. In that study, BTV cell-culture isolates were generated using only *Culicoides* and mammalian cell lines. In many instances, investigators still rely on the conventional propagation of BTV in ECE and then subsequent propagation in mammalian cells [14,15,16,17]. Currently, it is unclear if conventional propagation of BTV in ECE and mammalian cells results in similar attenuation of pathogenicity compared to propagation in *Culicoides* and mammalian cells. The genetic basis for the observed attenuation in the animal model is difficult to define due to the complexity of the multi-segment viral genome and variation in the levels of diversity within the quasispecies composition of the isolates studied [7,22].

The virulence determinants of BTV and cognate host receptors are yet to be identified. The entry of BTV into mammalian cells is precipitated by binding of viral protein 2 (VP2) to host cell receptor(s) with cell fusion and entry mediated by VP5 [23,24]. Binding to the insect midgut cell receptor however, predominantly relies on VP7 [25]. During virus replication, five non-structural (NS) proteins are produced. Synthesis of NS1 generates virus-specific tubules during active infection to support the virus translation machinery [26]. NS2 proteins are important for protecting viral RNA transcripts, regulating virus genome trafficking, and the formation of viral inclusion bodies [27,28]. Importantly, several non-structural proteins were shown to play a role in BTV pathogenicity. The host cell IFN response may be disrupted by NS3 and NS4 proteins [29,30]. The glycoprotein NS3/3a that is involved in virus egress during infection of insect cells [31] also possesses viroporin-like properties which can disrupt mammalian cellular permeability [32].

To evaluate the suitability of the chicken embryo model to study viral pathogenicity, we characterized the pathology and viral tropism in chicken embryos infected with the pathogenic virus strain BTV-3 Cyprus 1943 [33,34]. We compared *in ovo* infections caused by BTV from viremic sheep blood with that of virus passaged in cell cultures, either using a conventional propagation method (virus propagation in ECE followed by propagation in mosquito and mammalian cell lines) or a contemporary method (*Culicoides*-derived (KC) cell line followed by mammalian cell lines). To understand the genetic selection underlying changes of disease presentation, deep sequence analysis of the genome of inocula of cell-culture- and sheep-derived BTV quasispecies populations was also undertaken.

## 2. Materials and Methods

### 2.1. Ethics Statement

Animal experiments using ECE were approved by the Australian Animal Health Laboratory (AAHL) Animal Ethics Committee (AEC#1918) in accordance with the Australian National Health and Medical Research Council Code of Practice for the Care and Use of Animals for Scientific Purposes, Eighth Edition (2013).

### 2.2. Cell Lines and Viruses

A variant of baby hamster kidney cells (BHK-BSR) and African green monkey (Vero; ATCC^®^CCL-81^TM^) cells were grown in basal medium Eagle (BME) and minimum essential media (MEM) (Invitrogen, Melbourne, Australia), respectively, and supplemented with 5% fetal bovine serum (FBS) (Invitrogen), 2 mM glutamine (Invitrogen), and 100 IU/mL penicillin (Sigma, St. Louis, MO, USA). All mammalian cells were grown at 37 °C in a humidified incubator supplemented with 5% CO_2_. KC cells derived from *Culicoides sonorensis* were maintained in Schneider’s *Drosophila* media (Invitrogen) supplemented with 10% FBS and 100 IU/mL penicillin [35]. *Aedes albopictus* cells (C6/36) were grown in medium 199 (M199) (Invitrogen) supplemented with 10% FBS, 2 mM glutamine, and 100 IU/mL penicillin.

The reference virus strain of BTV-3 was obtained from the Onderstepoort Veterinary Institute, South Africa. It was determined by gene sequencing to be derived from the Cyprus 1943 strain (NCBI KP821039.1) [33,34]. This sheep blood-derived virus was previously demonstrated at AAHL to be highly pathogenic in sheep [36]. The virus was passaged a further four times in sheep, and the infectious sheep blood was stored in vapor-phase liquid nitrogen.

**Figure 1:**
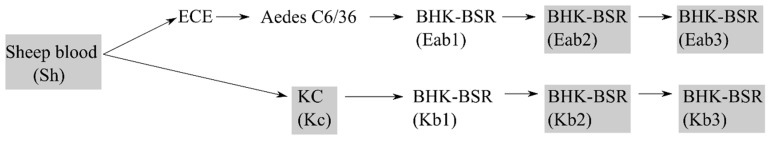
Passage history of the bluetongue viruses used in this study. The virus passages studied are in gray boxes and designated virus names are in brackets.

Virus isolation and propagation of the sheep-derived BTV-3 virus (Sh virus) was performed using two methods: (1) ECE (one passage), followed by *Aedes* cells (one passage), and then mammalian BHK-BSR cells (three passages), as recommended by the OIE [10]; (2) KC cells (one passage), followed by BHK-BSR cells (three passages) [7]. The isolates derived from ECE/Aedes/BHK-BSR were designated as Eab. For KC/BHK-BSR cell propagation, the isolates were designated as Kb; isolates Kb2 and Eab2 were derived from the second passage in BHK-BSR cells and Kb3 and Eab3 represent the third passage in BHK-BSR cells. Cell-culture-adapted BTV with two and three passages in mammalian cells were used in this study to represent low-passaged BTV used in published animal models of BT disease [7,16,17,18]. Sh virus passaged once in KC cells is hereafter referred to as Kc virus. The cell culture preparations and viruses used in this study are shown in Figure 1.

All viruses propagated in cell culture were prepared from supernatant harvested from infected cells following freezing at −80 °C and thawing at room temperature. The supernatants collected were titrated by serial 10-fold dilution and inoculated onto confluent Vero cell monolayers on 96-well plates. The cytopathic effect was visualized post-fixation with 0.1% methylene blue. The virus titer was determined using the Spearman–Karber method for calculation of median tissue culture infectious dose (TCID_50_) [37]. The virus isolates were stored at 4 °C for immediate use or −80 °C for later use.

### 2.3. Infection Studies in Chicken Embryos

Fertile chicken eggs were obtained from Turi Foods (Victoria, Australia). The virus inocula were BTV-3-infected sheep red blood cells lysed in sterile distilled water or cell-culture-passaged viruses diluted in MEM or Schneider’s media. For inoculation of ECE, a window on the eggshell overlaying a suitable blood vessel on the chorioallantoic membrane (CAM) was first cut and removed from 11-day-old ECE. One hundred microliters of virus or phosphate-buffered saline (mock infection) was then intravenously inoculated into the ECE using a 26G needle. The shell was sealed with cellophane tape and then incubated at 33 °C [38].

To study the time course of infection, four eggs infected with Sh virus (10^1.5^ TCID_50_) were randomly selected for humane euthanasia at one, two, and three days post infection (DPI). One uninfected age-matched ECE was randomly selected for humane euthanasia at the same time points as a negative control.

For the survival study, each virus was inoculated into six eggs. Viruses and doses inoculated into the eggs were as follows: cell-culture-passaged virus Eab2, Eab3, Kb2, and Kb3 (10^3.0^ TCID_50_ each), Kc virus (10^1.2^ TCID_50_), and Sh virus (10^1.5^ TCID_50_). The eggs were candled every 12 h after inoculation over the course of seven days. Upon the detection of embryo inactivity, the embryos were removed from the eggs immediately and humanely killed by decapitation. The embryo and CAM were then fixed in 10% neutral buffered formalin for 24 h.

### 2.4. Histopathology and Immunohistochemistry (IHC)

Whole ECEs were processed using routine histology methods and cut in after processing to allow thorough infiltration of paraffin to support the fragile tissues. Formalin-fixed tissues were then processed by standard histological methods, embedded in paraffin wax, and 4-μm sections were cut onto Flex IHC microscope slides (Agilent, Melbourne, Australia). Sections were then dewaxed through xylene and rehydrated with absolute alcohol, followed by 70% alcohol and water. To retrieve antigens, the sections were heated at 97 °C for 20 mins in a water bath (PT Link, Agilent) containing pH 9 buffer (EnVision™ FLEX target retrieval solution, Agilent). Sections were then rinsed in Tris wash buffer (0.05 mL/L Tris-HCl, 0.15 mol/L NaCl, 0.05% Tween-20, pH 7.5) (Agilent) and treated with 10% peroxidase-blocking agent (EnVision™ FLEX Peroxidase, Agilent) for 10 mins. The primary antibodies were diluted in Envision antibody diluent (Agilent) and incubated for 60 mins at room temperature (RT). The primary antibodies were used at the following dilutions: NS1 monoclonal antibody (mAb) at 1:1000, NS2 mAb at 1:6000, NS3 mAb at 1:800, VP7 polyclonal antibody at 1:1000, and active-caspase-3 at 1:2000 (Abcam, Melbourne, Australia). Sections labeled with mAb were further incubated with a secondary polymer (Envision Flex Mouse linker, Agilent) for 15 mins at RT. Subsequently, slides were then incubated with horseradish peroxidase (HRP)-conjugated secondary antibody (anti-rabbit and anti-mouse) (Agilent) for 20 mins at RT. The slides were washed with Tris buffer between each incubation step. Finally, AEC substrate (Agilent) was added onto the slides for 10 mins, counter-stained with Lillie Mayer’s hematoxylin (Australian Biostain, Australia), and cover slips were applied using aqueous mounting medium (Agilent). A duplicate serial tissue section was stained with hematoxylin and eosin stain for histological examination.

### 2.5. Assessment of the Immunohistochemical Labeling

The viral antigen load was assessed at high power field (hpf) of a light microscope at 40× magnification and qualitatively scored as abundant (score 3, more than 10 positive foci per hpf), moderate (score 2, between five and nine positive foci per hpf), minimal (score 1, between one and four positive foci per hpf), and undetectable (score 0). The operator (F.Z.X.L.) scoring proficiency was standardized against the scores achieved by the pathologist (J.Bd., J.B.). Images that were selected for the standardization process can be found in Figures S1 and S2 (Supplementary Materials). The slides were de-identified during the assessment and assessed by one operator (F.Z.X.L.). One representative field per organ was scored, and the scores were averaged for the study group to obtain mean values for each organ. The same assessments were also made for the active caspase-3 immunolabeling. Immunohistological scores were plotted as heat maps using ggplot v.3.1.0 [39].

### 2.6. One-Step Growth Curve Analysis

BTV-3 was added to triplicate wells of a 24-well plate containing Vero, BHK-BSR, or KC cells at a multiplicity of infection (MOI) of 0.01. Inoculated cells were incubated for 37 °C (Vero, BHK-BSR) or 28 °C (KC) for one hour. The inoculum was then removed, and cell monolayers were washed once with phosphate-buffered solution (PBS; 137 mM NaCl, 2.7 mM KCl, 10 mM Na_2_HPO_4_·2H_2_O, 1.8 mM KH_2_PO_4_, pH 7.3) and replaced with complete medium. Cells were incubated as above. Aliquots of cell-culture supernatant were collected from each well at 12 and 24 h post infection (HPI) and every 24 h thereafter until 96 HPI for mammalian cell culture, and up to 240 HPI for KC cells. The supernatants collected were titrated on Vero cells, as described above. Statistical analysis was performed on GraphPad Prism 5 by ANOVA followed by Bonferroni multiple-comparison test.

### 2.7. Deep Sequencing

Viral RNA was extracted from sheep blood (Sh virus) and virus harvested from cell-culture supernatant (Kc, Kb2, Kb3, Eab2, Eab3 virus) using the MagMAX™—96 Viral RNA Isolation Kit (Applied Biosystems, Foster City, CA, USA). The viral RNA was transcribed into complementary DNA (cDNA) and amplified with the SuperScript™ III One-Step RT-PCR system with Platinum^TM^ *Taq* high-fidelity DNA polymerase (Invitrogen) and gene-specific primers that were designed for highly conserved regions of the genomes (Table S1, Supplementary Materials). The cDNA was amplified on an Eppendorf Mastercycler with the following cycling parameters: 50 °C for 30 s, 95 °C for 2 mins, 40 cycles of 94 °C for 45 s, 50 °C for 60 s, and 68 °C for 60 s, followed by a cycle of 68 °C for 5 mins. An Illumina Nextera XT DNA library was prepared from purified DNA (Qiagen, Hilden, Germany) following the manufacturer’s instructions. Library DNA was quantified using the Invitrogen Qubit™ ds DNA HS assay and the average library size was determined on a Bioanalyzer using the Agilent High-Sensitivity DNA kit. Paired-end 150-bp sequencing was performed on an Illumina MiSeq instrument.

The raw reads from each sample were trimmed using Trimmomatic v.0.38 [40], trimming when the quality dropped below 3, and removing reads shorter than 75 bases. The cleaned reads were then aligned to a BTV-3 genome [34] using Hisat v.2.1.0 [41] with the default settings. The sequence alignment map (SAM) file was then manipulated using Samtools v.1.9.0, and new genomes were assembled by a combination of BCFtools and vcfutils [42]. Shannon-entropy analysis [43] was performed using custom scripts in Python v.3.5.2. These scripts used pysam v.0.15.0 (https://github.com/pysam-developers/pysam) to analyze the alignment files and the following Shannon entropy equation:

$$H_{s}\left(p\right)=-\overset{M}{\underset{i=1}{\sum }}P_{i}\log_{2}\left(P_{i}\right)$$

where *P_i_* is the frequency of an individual genotype, and *M* is the number of clones sequenced. *H_s_* values range from 0 (completely homogeneous) to 2 (completely heterogeneous) [43]. Plots of the Shannon entropy values were created using ggplot v.3.1.0 [39]. Raw sequencing data are available in the Sequence Read Achieve under the accession numbers SRR8868283 to SRR8868288.

## 3. Results

### 3.1. Sheep Blood Containing BTV-3 is Pathogenic in the ECE Model

**Figure 2:**
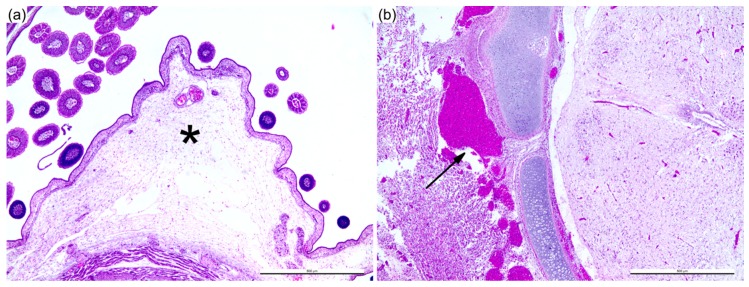
Pathology of Bluetongue virus (BTV)-infected chicken embryos at three days post infection (DPI). Expansion of subcutaneous space consistent with edema (**a**). Hemorrhage at the occipital region of the cranium (**b**). Scale bar = 500 μm.

In order to characterize the pathogenesis of BTV in ECE, we performed a time-course study using sheep blood containing BTV-3 (Sh virus; Figure 1). The progression of the infection was characterized by evaluation of apoptosis and BTV dissemination in the ECE by IHC. At one and two days post infection (DPI), the BTV-infected embryos were not clinically affected, nor was significant gross or microscopic pathology detected. At 3 DPI, the infected ECEs were all dead. On gross examination, the skin of the embryo appeared bright red as a result of vascular congestion and hemorrhage. Expansion of the subcutaneous space was also observed during the examination, which was confirmed by histopathological analysis to be consistent with edema (Figure 2a). Hemorrhage was also observed at the back of the skull (Figure 2b) and in appendages of the embryo.

**Figure 3:**
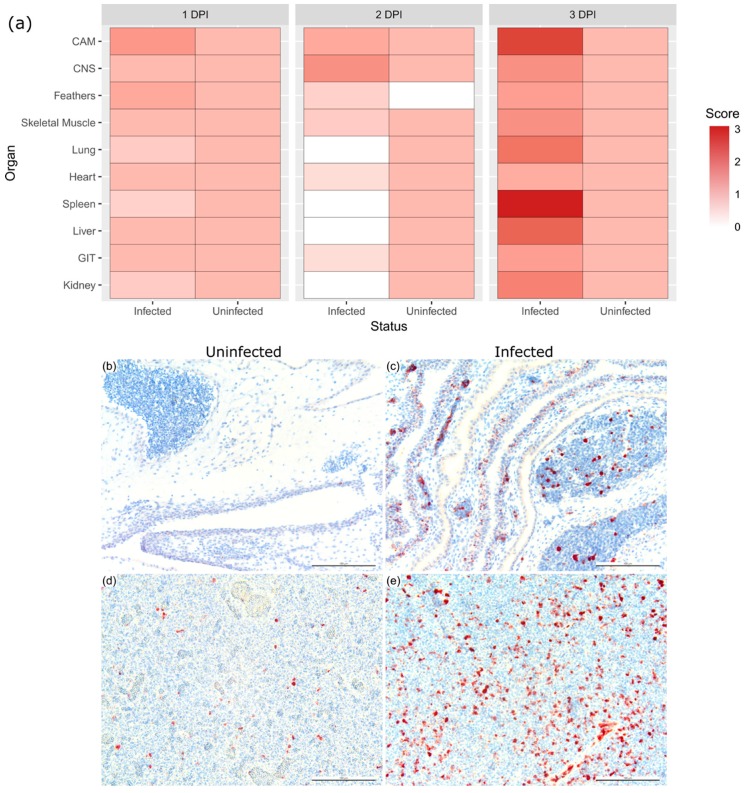
Comparison of expression of activated caspase-3 (CASP3) in three-day-old BTV-infected chicken embryos. Score of activated caspase-3 labeling in the different chicken embryo organ systems at days 1, 2, and 3 after BTV infection and in uninfected age-matched embryos (**a**). Chorioallantoic membrane (CAM) of uninfected (**b**) and infected (**c**) and spleen of uninfected (**d**) and infected (**e**). Staining scores: 3 = abundant, 2 = moderate, 1 = minimal, 0 = undetectable. CNS = central nervous system; GIT = gastrointestinal tract. Scale bar = 100 μm.

To investigate if the pathogenic outcome *in ovo* was a result of apoptosis, tissue sections of infected ECE and uninfected age-matched ECE were immunolabeled with activated caspase-3 (CASP3), a cellular indicator of apoptosis (Figure 3). At 1 and 2 DPI, there was minimal CASP3 labeling in both the infected and uninfected ECEs, reflecting the normal turnover of cells in the embryonic developmental stage [44]. On the third day of infection, there was increased activation of CASP3 in various organs of the infected ECE, with significant amount of labeling in the CAM and spleen (Figure 3a). In the CAM, activated CASP3 could be detected in areas such as the chorionic epithelium, endothelial cells of both macro- and micro-vasculature, and the hematopoietic cells (Figure 3c). The splenic cells of the red pulp and trabeculae of the spleen were also strongly immuno-positive for CASP3 (Figure 3e). These results indicate that embryo death was associated with increased apoptosis.

**Figure 4:**
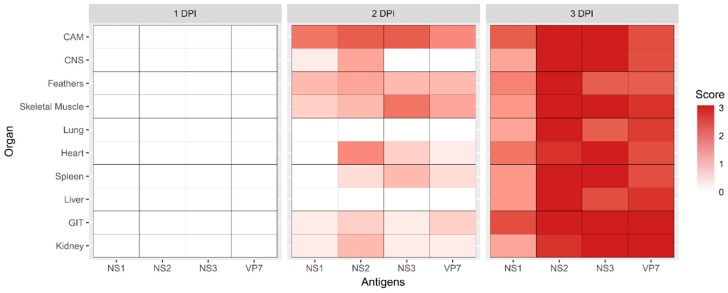
The abundance of viral antigens and their distribution, plotted as heat maps, in various organs of the chicken embryo at days 1, 2, and 3 post infection. Viral antigen loads were assessed semi-quantitatively at 40× magnification. Scores: 3 = abundant, 2 = moderate, 1 = minimal, 0 = undetectable. CAM = chorioallantoic membrane; CNS = central nervous system; GIT = gastrointestinal tract.

The dissemination of the BTV-3 Sh virus in the embryo was evaluated using IHC specific for each of the BTV antigens—NS1, NS2, N3/3a, and VP7 (Figure 4). No virus antigen was detected on 1 DPI. Two days after virus inoculation, moderate amounts of BTV antigens were apparent in the CAM. Low amounts of antigens were detectable in most organs, but not in the lungs and liver. On the third day of infection, antigens were diffusely distributed across various organs within the ECE. The abundance of BTV antigens detected on 3 DPI coincided with the activation of apoptosis.

**Figure 5:**
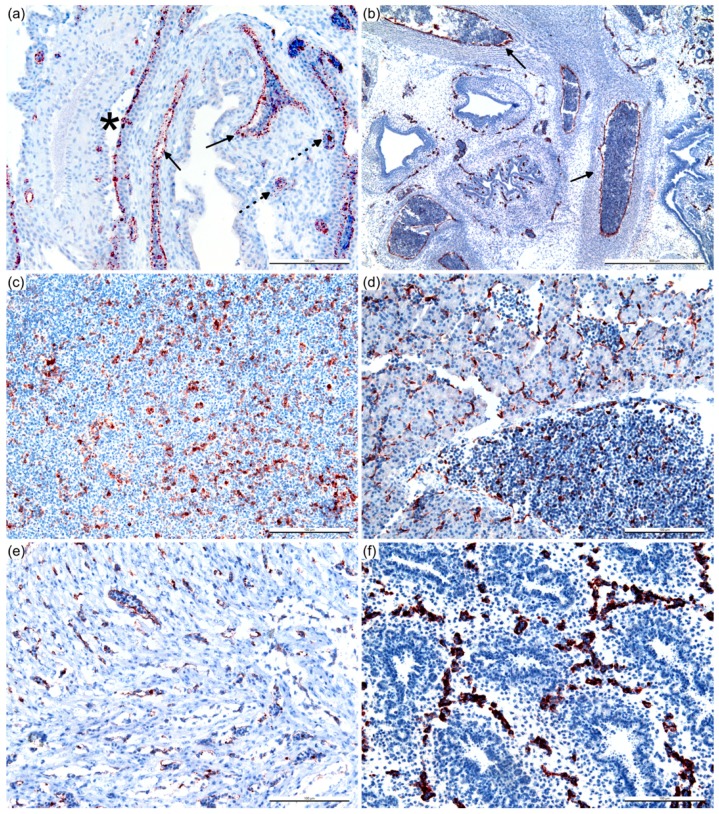
BTV non-structural protein 2 (NS2) antigens in infected chicken embryo at 3 DPI. Immuno-positive staining (red/brown color) in the chorionic epithelium (asterisk) and macro- (arrow) and microvascular (dashed arrow) endothelial cells of the chorioallantoic membrane (**a**), endothelium of the aorta and vena cava (arrow) (**b**), splenic cells (**c**), liver (endothelium of the central vein and sinusoidal endothelium, and hematopoietic cells) (**d**), and microvasculature of the heart (**e**) and lung (**f**). Immunohistochemistry, with hematoxylin counterstain. Scale bar = 500 μm (**b**), 100 μm (**a**,**c**–**f**).

In the CAM, BTV antigens were detected in the chorionic epithelium and macro- and microvascular endothelial cells (Figure 5a). Endothelial cells in the aorta and vena cava were also immuno-positive for BTV (Figure 5b). Abundant BTV antigens were present among the splenic cells (Figure 5c), and within the liver (endothelium of the central vein and sinusoidal endothelium, and hematopoietic cells) (Figure 5d), microvasculature of the heart (Figure 5e), and lung (Figure 5f). Generally, multiple antigen detection identified vascular-specific BTV replication.

**Figure 6:**
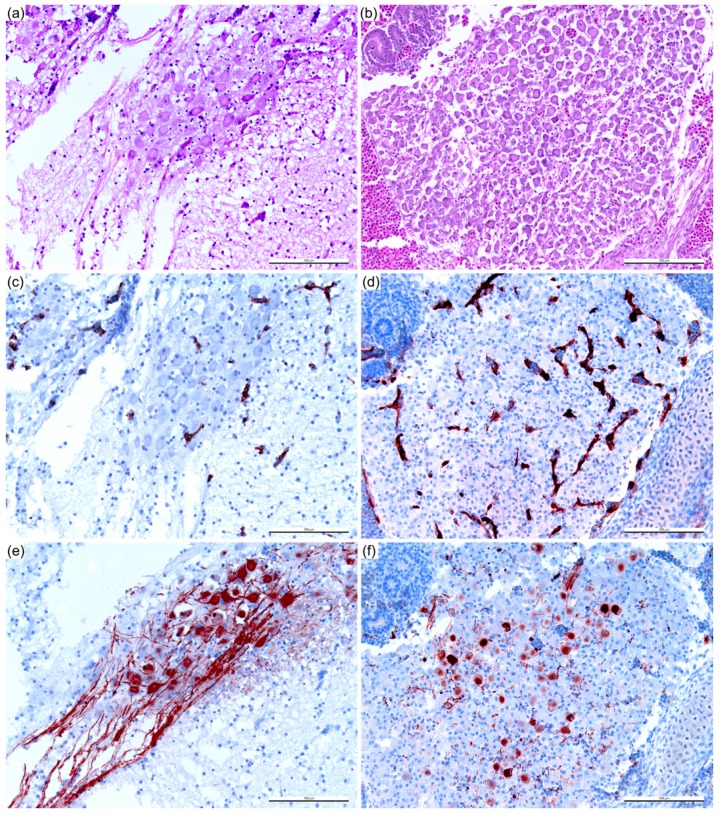
Neuronal and vascular replication of BTV within cerebrum (**a**,**c**,**e**) and dorsal root ganglion (**b**,**d**,**f**). Serial sections of the cerebrum (**a**,**c**,**e**) and dorsal root ganglion (**b**,**d**,**f**) were stained with hematoxylin and eosin (H&E) (**a**,**b**) and immunohistochemistry (IHC) for NS2 (**c**,**d**) and NS1 (**e**,**f**), showing NS2 antigens within vascular endothelial cells (**c**,**d**) and NS1 antigens within the neuronal cells (**e**,**f**) of the cerebrum and dorsal root ganglion, respectively. Vascular localization of NS2 antigens was similar to that of NS3/3a and viral protein 7 (VP7) (not included in the panel). Scale bar = 100 μm.

In the cerebrum, NS2 (Figure 6c), NS3/3a, and VP7 were present in the vasculature without significant histopathological lesions observed (Figure 6a). Unlike other BTV antigens detected in the cerebrum, the low amount of NS1 detected was attributable to the immuno-labeling only in the neuronal cells (Figure 6e), and the positive neuronal cells were randomly and sparsely distributed. Similar immunolabeling patterns were occasionally observed within the spinal dorsal root ganglion (Figure 6d,f). The presence of BTV antigens was not observed in any uninfected ECE.

Our findings indicated that the strain of BTV-3 used was able to replicate in the chicken embryo, as determined by the presence of diffuse viral antigen presence in the vascular and neuronal systems. The disease in the chicken embryo was most likely a combination of viral replication in the vascular bed and the brain, accompanied by the widespread activation of programmed cell death in the organs, leading to acute vascular dysfunction, multi-organ failure, and death.

### 3.2. BTV Propagation Method Affects Replicative Fitness In Vitro

**Figure 7:**
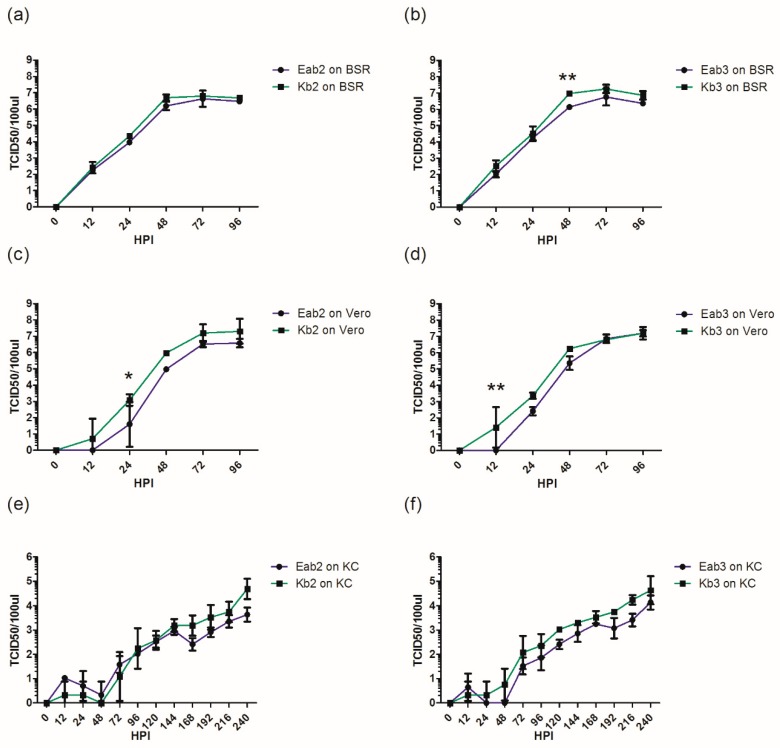
One-step growth analysis of second and third passaged BTV on mammalian baby hamster kidney cells (BHK-BSR) (**a**,**b**), Vero (**c**,**d**), or *Culicoides*-derived (KC) cells (**e**,**f**). Cells were infected at a multiplicity of infection (MOI) of 0.01 in triplicate. Supernatants were collected at 12 h, 24 h, and then every 24 h until 96 (BSR/Vero) or 240 (KC) h post infection (HPI) and then titrated in Vero cells using limiting dilution analysis. The error bars represent standard deviation. Statistical analysis was performed using ANOVA followed by Bonferroni multiple-comparison test. * *p* < 0.05, ** *p* < 0.01. Kb = KC/BHK-BSR; Eab = embryonated chicken egg (ECE)/*Aedes*/BHK-BSR.

To determine if the different cell passage histories of BTV, either via conventional (Eab) or contemporary (Kb) propagation (Figure 1) affected their replicative fitness in vitro, one-step growth curve analyses were performed using BHK-BSR and KC cells, as well as Vero cells, representing an additional mammalian cell line known to be permissive for BTV replication [7]. While the growth kinetics and endpoint titers of the Eab and Kb viruses evaluated in BSR cells were similar (Figure 7a,b), Vero cells infected with Kb2 and Kb3 were able to produce infectious BTV as early as 12 h post infection (HPI), whereas detectable titers of Eab2 and Eab3 were not observed until 24 HPI (Figure 7c,d). While not statistically significant, Kb-infected KC cells were also able to produce slightly more infectious virus than the Eab viruses (Figure 7e,f). These results indicated that sheep-derived BTV-3 passaged in KC cells followed by BHK-BSR cells have a replicative advantage over virus passaged in ECE, followed by *Aedes* and BHK-BSR cells.

### 3.3. Reduction of Pathogenicity In Ovo with Cell-Culture-Passaged BTV

**Figure 8:**
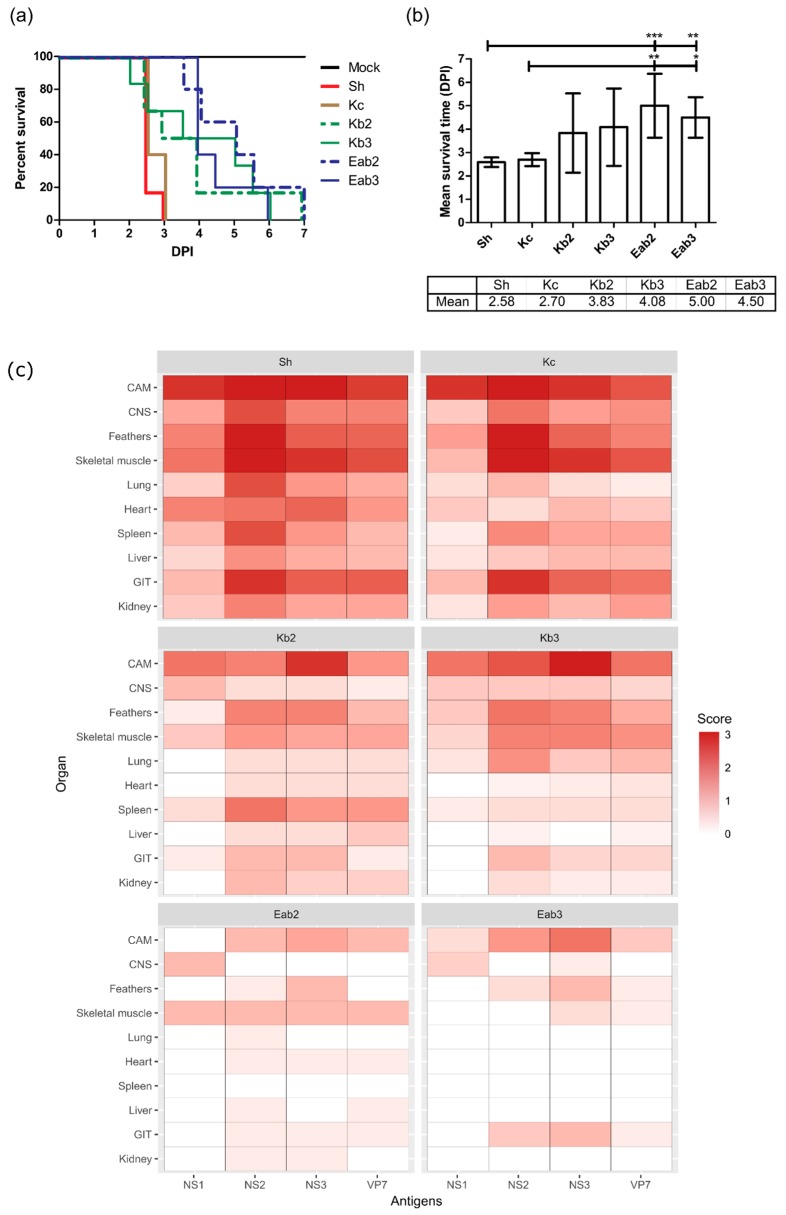
Survival curves (**a**) and mean survival times (**b**) of the ECEs infected with sheep blood BTV (Sh) and cell-culture-passaged BTV (Kc, Kb2, Kb3, Eab2, Eab3). Statistical analysis was performed using ANOVA followed by Bonferroni multiple-comparison test. * *p* < 0.05, ** *p* < 0.01, *** *p* < 0.001. The viral antigen loads from tissues collected at clinical endpoint were assessed semi-quantitatively by light microscopy at 40× magnification, and the average scores for each organ were plotted as heat maps (**c**). Scores: 3 = abundant, 2 = moderate, 1 = minimal, 0 = undetectable. CAM = chorioallantoic membrane; CNS = central nervous system; GIT = gastrointestinal tract. Kb = KC/BHK-BSR; Eab = ECE/*Aedes*/BHK-BSR.

To determine the effect of viral passage through different cell culture systems in ECE, we next compared the pathogenicity of BTV-3 derived from sheep blood (Sh) with cell-culture-passaged virus. Pathogenicity in this model was defined by the duration of survival over the seven days following infection. The cell-culture preparations and viruses used in this study are shown in Figure 1. All the ECEs inoculated with BTV-3 Sh and Kc viruses died as a result of BTV infection within 3 DPI (Figure 8a), whereas other ECEs infected with Eab or Kb viruses reached 100% mortality between 6 and 7 DPI (Figure 8a). No mortalities were observed in mock-infected ECE. The average survival times of embryos infected with Sh and Kc viruses were lower than those infected with Kb viruses, which were in turn lower than those infected with Eab viruses (Figure 8b); however, only the differences between Sh and Kc versus Eab2 and Eab3 were statistically significant.

The ECEs harvested at the clinical endpoints were subjected to BTV IHC staining and analysis (Figure 8c). There was a significant reduction of viral antigen load among ECEs infected with Eab and Kb viruses compared to embryos infected with Sh and Kc viruses. BTV antigens were widely distributed in various organ systems among the Sh- and Kc-infected ECE. In contrast, virus antigens were primarily found within the CAM, the gastrointestinal tract (GIT), and peripheral tissues such as feathers and skeletal muscles for ECEs infected with Eab and Kb viruses. BTV antigens were slightly more abundant among the Kb virus-infected ECEs compared to Eab-infected ECEs, suggesting different levels of replication efficiency. However, the differences observed *in ovo* were not significant.

Taken together, these observations demonstrated that the BHK-BSR-passaged BTVs were less pathogenic in the chicken embryo compared to viremic sheep blood and BTV that was passaged once in KC cells, despite the lower titers of Sh and Kc viruses used for inoculation.

### 3.4. Gene Segment-Specific Mutations and Virus Diversity within BTV Populations

As the cell-culture-propagated BTV produced an attenuated pathotype in ECEs, we sought to determine if the genetic diversity present in each of these virus cultures was associated with the observed pathogenicity. Selected genome segments (Seg-2, -5, -6, -7, -8, -9, and -10) of each of the virus inocula were amplified by PCR using BTV-3-specific primers directed at highly conserved regions within each segment, followed by deep sequencing. On average, 1.7 million raw reads were produced for each virus sequenced, with a 99.49% alignment rate to the reference genome [34] and an average read depth of 5975.

Non-synonymous and synonymous nucleotide mutations were detected among the consensus sequences of Seg-2, -5, -6, -8, and -9 in the cell-culture-propagated BTV in comparison to the Sh virus (Table 1). In Kb viruses, there was one synonymous mutation and three non-conservative amino-acid mutations among the five non-synonymous changes, whereas Eab had one synonymous mutation, with three non-conservative amino-acid mutations among the four non-synonymous mutations. No mutations were found in the consensus sequences of Seg-7 and -10 for any of the viruses, reflecting the conservation of these genome segments within the BTV species [7]. Mutations that were present commonly among Eab and Kb viruses were in Seg-8 (Q169R) and Seg-9 (M5I). Cell-culture-passage-specific mutations were also identified. Eab viruses harbored unique amino-acid mutations in VP2 (G827E, body domain of VP2 [24]) and NS1 (E94G, body domain of NS1 [26]). As for Kb viruses, unique mutations were found in VP5 (T186I, unfurling domain [23]), VP6 (T33M), and NS4 (R3K) (Table 1).

**Figure 9:**
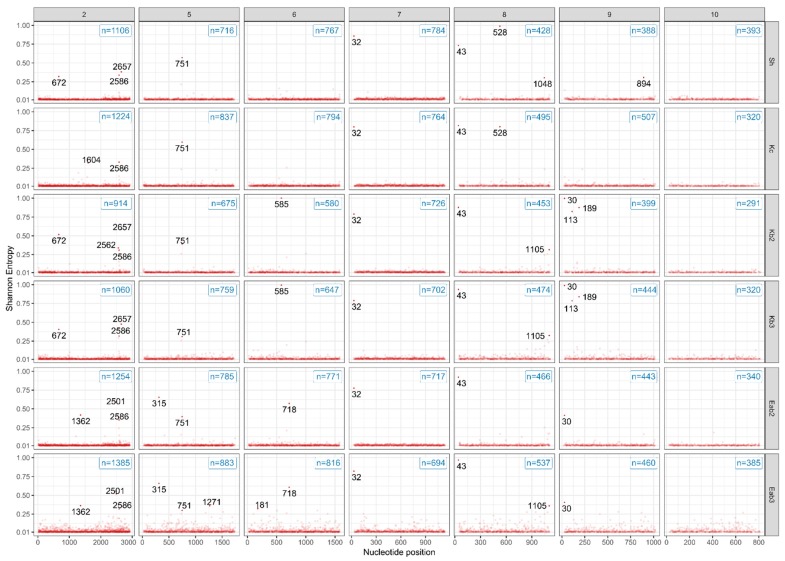
Shannon-entropy analysis of BTV-3 from infectious blood and cell-culture-passaged viruses. Deep sequencing was performed on amplicons of the genome segments shown. The *y*-axis represents nucleotide positions and the *x*-axis represents Shannon-entropy values. Nucleotide positions are indicated by a pink dot if the Shannon entropy value for that position is >0.3. Total count of nucleotide positions achieving entropy >0.01 are shown in the boxes on the top right. Kb = KC/BHK-BSR; Eab = ECE/*Aedes*/BHK-BSR.

The nucleotide sequence variability [45] of each virus inoculum was examined by Shannon-entropy analysis (Figure 9). Examination of nucleotide positions harboring entropy values >0.3 revealed cell-culture-passage-specific differences in genomic diversity. Both the Eab and Kb viruses shared nucleotide variation in Seg-9 (position (pos.) 30). Among the Eab viruses, unique nucleotide diversities were noted in Seg-2 (pos. 1362), Seg-5 (pos. 315), and Seg-6 (pos. 718), whereas the Kb viruses acquired diversity in Seg-6 (pos. 585), Seg-8 (pos. 1105), and Seg-9 (pos. 113 and 189). The nucleotide variability from Seg-2, -5, -6, -8, and -9 was found to correspond to the nucleotide mutations observed in the consensus sequence (Table 1). There was loss of a sheep-specific variant in Seg-8 (pos. 528), and also re-emergence of sheep-specific nucleotide variants in Seg-2 (pos. 672 and 2657) in the Kb viruses after dropping below the entropy threshold following propagation in KC cells.

We further evaluated the genetic diversity by calculating the percentage of nucleotide positions which achieved >0.01 entropy for each genomic segment (Table 2). A higher level of genetic diversity was observed for Kc virus compared to Sh, Kb2, and Eab2 viruses in Seg-5, -6, -8, and -9, which is similar to the findings of an earlier report [7]. For Kb viruses, a decrease in genetic diversity among Seg-2, -5, -6, -7, and -10 was observed following the second passage in mammalian cells (Kb2) compared to the stock Sh virus, whereas reduction in genetic diversity in all segments was observed in relation to the parental Kc virus. This was followed by an increase in diversity after the third passage in mammalian cells in all segments except Seg-7, in which there was a slight reduction. In contrast Eab viruses showed an increase in genetic diversity compared to parental Sh virus in Seg-2, -5, -6, -8, and -9, whereas a decrease was found in Seg-7 and -10. As for Kb viruses, a third passage of Eab in mammalian cells led to an increase in diversity in all segments except Seg-7. This corresponds to our earlier finding of no mutations in the consensus sequence of Seg-7 in any of the viruses analyzed (Table 1). Interestingly, the highest levels of percentage diversity found in Seg-2, -5, -6, and -8, across all viruses analyzed, occurred in Eab3, which experienced the highest number of propagation steps.

## 4. Discussion

The use of ECEs for BTV isolation was first developed in the 1940s [46] and is adopted worldwide for the isolation of BTV and other orbiviruses including AHSV. The pathogenesis of AHSV *in ovo* was also described in an earlier study [11].

In this study, BTV infection of the ECE was first detected two days after virus inoculation, with significant virus replication in various organs except the liver and lung of the developing chicken embryo. This suggested that organs such as CAM, skeletal muscle, heart, spleen, GIT, and kidneys are highly permissive organs for early virus replication. The lack of virus replication in the liver and lung could be due to shunting of venous blood of the ECE at the embryonic development phase [47]. Despite this, the dissemination of BTV to various organs within the chicken embryo is similar to the conventional intravenous challenge approach in sheep [20].

The vascular pathology in the ECE, as characterized by hemorrhage, congestion, and edema, was associated with the increased activation of CASP3, which was observed within the microvascular endothelium and hematopoietic cells of various organ systems. The primary pathology *in ovo* was likely damage to the CAM, as it is a critical organ for gaseous exchange in the developing embryo [48]. Splenic cells could have contributed to the release of inflammatory mediators from the type I and III IFN systems during embryogenesis [49]. Nevertheless, the immune response mechanism was not examined in this study.

In general, BTV infection *in ovo* was similar to earlier investigations using AHSV, which showed that significant amounts of viral antigens could be detected, along with embryo mortality, within 3 DPI with a similar tissue tropism profile [11]. The extent of BTV replication in the skin, lung, spleen, liver, and kidney was also similar to experimental infection in sheep [13,50]. Nonetheless, in the present study, BTV replication was detected in both the macro- and microvasculature as confirmed by the detection of multiple BTV antigens. Macrovascular tropism was not reported in the ECE infected with AHSV [11], nor was it a feature in the sheep model of infection [50]. It is possible that during embryonic development, the endothelial cells retain progenitor features that could be common throughout the vascular tree [51] and that these are highly permissive for BTV infection. In an earlier *in ovo* study of BTV, in situ hybridization of Seg-3 RNA (encoding VP3) detected BTV replication within the renal and bronchial epithelial cells of the ECE [12]. Epitheliotropism of BTV within the renal and respiratory systems was not observed in our study based on the detection of structural protein VP7. This observation was also not reported in the AHSV infection model [11]. We suspect that the differences observed could be attributed to the detection method (RNA versus antigens) and the passage method to generate virus inoculum.

In addition to endotheliotropism, BTV replicated within the neuronal cells in the ECEs. Similar NS1 neuronal staining was observed among ECEs infected with both the cell-culture-passaged viruses and sheep-derived viruses tested in this study. In the earlier study of BTV *in ovo*, RNA Seg-3 (encoding the structural protein VP3) was found in the granular cells of the cerebellum [12], whereas, in our study, the only structural protein antigen we examined, VP7, was only detectable in vascular endothelial cells. In a different study, the Seg-6 (VP5) was purportedly a determinant for neurotropism of a virulent isolate of BTV-11 in a mouse model [52,53]. Neurotropism was also reported in other naturally occurring orbiviral infections, for example, Wallal virus in kangaroos [54], Peruvian horse sickness virus and Elsey virus in horses [55], and laboratory-adapted AHSV [56,57]. The use of a BTV modified live vaccine (MLV) is contraindicated for pregnant ruminants, as neurological congenital deformity associated with this practice was reported globally [6]. Moreover, the capacity for an endemic *Culicoides* vector species to transmit MLV strains in the field, especially with their potential for neurotropism [58], makes the use of MLVs for BT disease control quite problematic. By specifically visualizing BTV antigens NS1, NS2, NS3/3a, and VP7, this model demonstrated both the endotheliotropism and neurotropism of BTV. Nevertheless, it was not an exhaustive exploration of IHC applied to study BTV pathogenesis. Other BTV proteins should also be incorporated in future studies to evaluate their specific roles in the pathogenesis of BTV.

Passaging BTV in vitro using different propagation methods could impose different selection pressures, potentially impacting virus replicative fitness and pathogenicity. In this paper, we compared the conventional Eab method and the contemporary Kb method using KC cells. Although no significant differences in replicative fitness were observed in KC or BHK-BSR cells in vitro, virus produced using the Kb method displayed more rapid replication in the early stages of propagation in Vero cells and higher titers, compared to Eab viruses. This indicates a difference in the replication of these viruses in mammalian cell lines derived from different species, which was reflected in the differences observed in mean survival times and antigen loads in ECE (Figure 8).

Arboviruses with a serial passage history in mammalian cell culture can have a reduced ability to induce disease in animals [7,59]. As demonstrated with Venezuelan equine encephalitis virus (VEEV), cell-culture-adapted virus induced an attenuated disease phenotype in a rodent model [60]. This was attributed to the acquisition of heparin sulfate binding ability, which in turn contributed to the rapid clearance of VEEV in vivo [60]. For BTV, the outer capsid protein VP2 (encoded by Seg-2) is involved in attachment to mammalian cells. In our study, increased levels of genetic diversity, along with an amino-acid mutation, were found for Seg-2 of the Eab viruses. It is, therefore, possible that there was preferential selection of a phenotypic variant for the mammalian host cell receptor-binding protein for these viruses. We also showed that the genetic diversity of Seg-7 (encoding VP7) was reduced over the course of in vitro passages and could be associated with the attenuation of pathogenicity *in ovo*. VP7 is known to mediate binding to the insect cell midgut membrane receptor [25], but its interaction with mammalian host cells is unclear. Serial passage of BTV in permissive mammalian cells could potentially reduce the requirement for attachment to insect cell receptors, thereby leading to a concomitant attenuation in pathogenicity *in ovo*. Therefore, we propose that the genetic selection for receptor binding during virus propagation in vitro has important implications for virus pathogenicity in mammalian hosts.

As replication of RNA viruses is reliant on the error-prone viral RNA-dependent RNA polymerase (RdRp), RNA viruses exist as genetically diverse viral populations (quasispecies) [61,62]. Consistent with previous findings [7], we observed that the passage of animal-derived BTV in the *Culicoides* KC cell line led to an increase in the genetic diversity of several viral genome segments (Table 1 and Table 2; Figure 9). Among other arboviruses, the innate immunity of insect cells mediated by RNA interference (RNAi) was reported to promote genetic diversity [63]. BTV-infected KC cells can also mount an antiviral RNAi response [64]. Therefore, we speculated that this form of immunity in KC cells could facilitate the selection of elevated levels of variation within BTV quasispecies.

The attenuation of BTV pathogenicity is linked to the evolution of viral quasispecies [7]. We observed an increase in the genetic diversity of Seg-2, -5, -6, -8, and -9 following virus propagation in KC cells or serial passage in ECE/*Aedes*/BHK-BSR cells. However, when KC cell-passaged virus was further propagated in BHK-BSR cells, a loss of diversity was observed within all segments. After a third passage in BHK-BSR cells, increased levels of diversity were observed in all segments (except Seg-7) as the viruses adapted to these cells. In a different study, the adaptation of Chikungunya virus (CHIKV) to a permissive vertebrate host cell environment led to attenuation and increased genetic diversity due to the accumulation of non-viable mutants [65], which is consistent with our findings. The trend observed in Seg-7, however, was in contrast to a previous study where there was a modest increase in genetic diversity in BTV that was propagated by up to two passages only in mammalian cell lines [7]. To avoid introducing variant selection and bias through laboratory-based cell culture [20,66], the induction of a reliable BT disease in infected animals ideally requires the use of viremic sheep blood as inoculum [7]. As virus stocks derived from ruminants might not be readily available in all institutions, this could be a barrier to study BTV in vivo. Consequently, BTV with low-passage history in mammalian cell culture is often used in animal models of BT disease [7,16].

We observed several nucleotide mutations that were common to both Eab and Kb viruses, but specific mutations were also present in Eab and Kb viruses. Non-synonymous mutations in VP2 and NS1 were identified for the Eab viruses, and in VP5 and VP6 for the Kb viruses, potentially affecting virus entry (VP2, VP5) and replication efficiency (NS1, NS4, VP6). Nucleotide mutations detected within the encoding regions for NS2 (Q169R) and VP6 (M5I) were common among cell-culture-passaged viruses and could be related to the attenuation of virus phenotype. Quasispecies analysis revealed generally lower levels of genetic diversity of Kb viruses in most genomic segments examined, following passage in KC cells, similar to an investigation on alternatively passaged CHIKV in *Aedes* and mammalian cells [65]. Different selection pressures may be expected to be imposed in each of these passages, as the virus population adapts to a clonal midge cell line followed by a clonal rodent cell line. Virus samples following propagation in either ECE or *Aedes* cells were not available for analysis in this study. However, it can also be expected that BTV is subjected to even more diverse selective pressures within a complex avian culture system, followed by clonal mosquito cells and clonal rodent cells.

In this study, high-throughput sequencing of amplicons derived from BTV genome segments was used to acquire sufficient sequencing depth to evaluate the extent of potential quasispecies generated diversity. Direct sequencing of viral RNA was also attempted in this study. However, this was problematic for samples from sheep blood, as the read coverage was inadequate for analysis. The prior production of viral amplicons by PCR for next-generation sequencing could potentially introduce bias through the amplification of particular gene-specific segment regions. To address this, we designed PCR primer sets targeting conserved regions of the BTV-3 genome, based on available BTV genome sequences in the NCBI database. Primer sets were used to amplify overlapping amplicons to minimize potential primer bias, and high-fidelity enzymes were incorporated into the PCR. We performed rigorous post-sequencing processes to remove poor-quality reads and selected genome coverage >100×. We also performed Shannon-entropy analysis that was independent of the reference or consensus genome. Another potential limitation of this study was that Seg-1, -3, and -4 were not included in our analyses. Given that these segments are highly conserved across the BTV serogroup [67] and previous studies did not generally implicate determinants on these genes being of major significance in virus pathogenicity [22,68], we concentrated our resources on the remaining genome segments.

Herein, we report on an alternative laboratory animal model for BTV that is permissive to both clinical-derived and cell-culture-passaged BTV and that can be readily adopted in most microbiological laboratories. The findings of this study strongly suggested that different methods of virus propagation impose different selection pressures on BTV populations and impact upon the nature of virus-induced pathogenicity. The altered viral population diversity and nucleotide mutations among structural and non-structural proteins that we observed as a result of different passage regimens led to an attenuated *in ovo* phenotype that may translate to attenuation in sheep or other ruminant hosts of BTV. Future in vivo investigations will be required to confirm the *in ovo* findings. Our findings support the recommendations of a previous study [7] that caution should be exercised when selecting virus inoculum for disease modeling.

## Figures and Tables

**Table 1:** Nucleotide and amino-acid mutations found in genome segments of cell-culture-passaged viruses in comparison to analogous sequences of the BTV-3 from infectious blood (Sh).

Segment (Protein)	Sh	Kc	Kb2	Kb3	Eab2	Eab3
**2 (VP2)**	-	-	-	-	G2501A **(G827E)**	G2501A **(G827E)**
**5 (NS1)**	-	-	-	-	A315G **(E94G)**	A315G **(E94G)**
**6 (VP5)**	-	-	C585T **(T186I)**	C585T **(T186I)**	A718G	A718G
**7 (VP7)**	-	-	-	-	-	-
**8 (NS2)**	-		A528G **(Q169R)**	A528G **(Q169R)**	A528G **(Q169R)**	A528G **(Q169R)**
**9 (VP6)**	-	-	A30G (M5I)	A30G (M5I)	A30G (M5I)	A30G (M5I)
			C113T **(T33M)**	C113T **(T33M)**		
			G189A	G189A		
**9 (NS4)**	-	-	G189A (R3K)	G189A (R3K)	-	-
**10 (NS3/3)**	-	-	-	-	-	-

Non-synonymous mutations are indicated in brackets and non-conservative mutations are emphasized in **bold** font. VP = viral protein; NS = non-structural protein; Kc = *Culicoides*-derived; Kb = KC/baby hamster kidney cells (BHK-BSR); Eab = embryonated chicken egg (ECE)/*Aedes*/BHK-BSR.

**Table 2:** Genetic diversity of BTV-3 from infectious sheep blood and cell-culture-passaged viruses.

	Sh	Kc	Kb2	Kb3	Eab2	Eab3
**Seg-2**	52.9 *	58.6	43.7	50.7	60.0	66.3
**Seg-5**	40.8	47.7	38.5	43.3	44.8	50.4
**Seg-6**	55.2	57.1	41.7	46.5	55.5	58.7
**Seg-7**	68.6	66.8	63.5	61.4	62.7	60.7
**Seg-8**	40.7	47.1	43.1	45.1	44.3	51.0
**Seg-9**	37.9	49.6	39.0	43.4	43.3	45.0
**Seg-10**	50.1	40.8	37.1	40.8	43.3	49.0

* Percentage diversity = $\frac{\mathrm{Number}\;\mathrm{of}\;\mathrm{nucleotide}\;\mathrm{positions}\;>\;0.01\;\mathrm{entropy}\;}{\mathrm{Length}\;\mathrm{of}\;\mathrm{genomic}\;\mathrm{segment}\;}\times 100$. Seg = segment; Kb = KC/BHK-BSR; Eab = ECE/*Aedes*/BHK-BSR.

## Supplementary Materials

The following are available online at https://www.mdpi.com/1999-4915/11/5/481/s1: Figure S1. Characteristics of the BTV IHC labeling; Figure S2. Scoring of BTV IHC; Table S1. List of primers used for generation of BTV amplicons.
