# Supplementary material for: Attenuation of Bluetongue Virus (BTV) in an in ovo Model Is Related to the Changes of Viral Genetic Diversity of Cell-Culture Passaged BTV
Source: Viruses. 2019 May 26;11(5):481. doi: 10.3390/v11050481 (PMC6563285; doi:10.3390/v11050481)
Supplement: Supplementary file 1 [file viruses-11-00481-s001.zip › Supplementary File 1 IHC images and PCR primers.docx]

**Supplementary Figures**

**
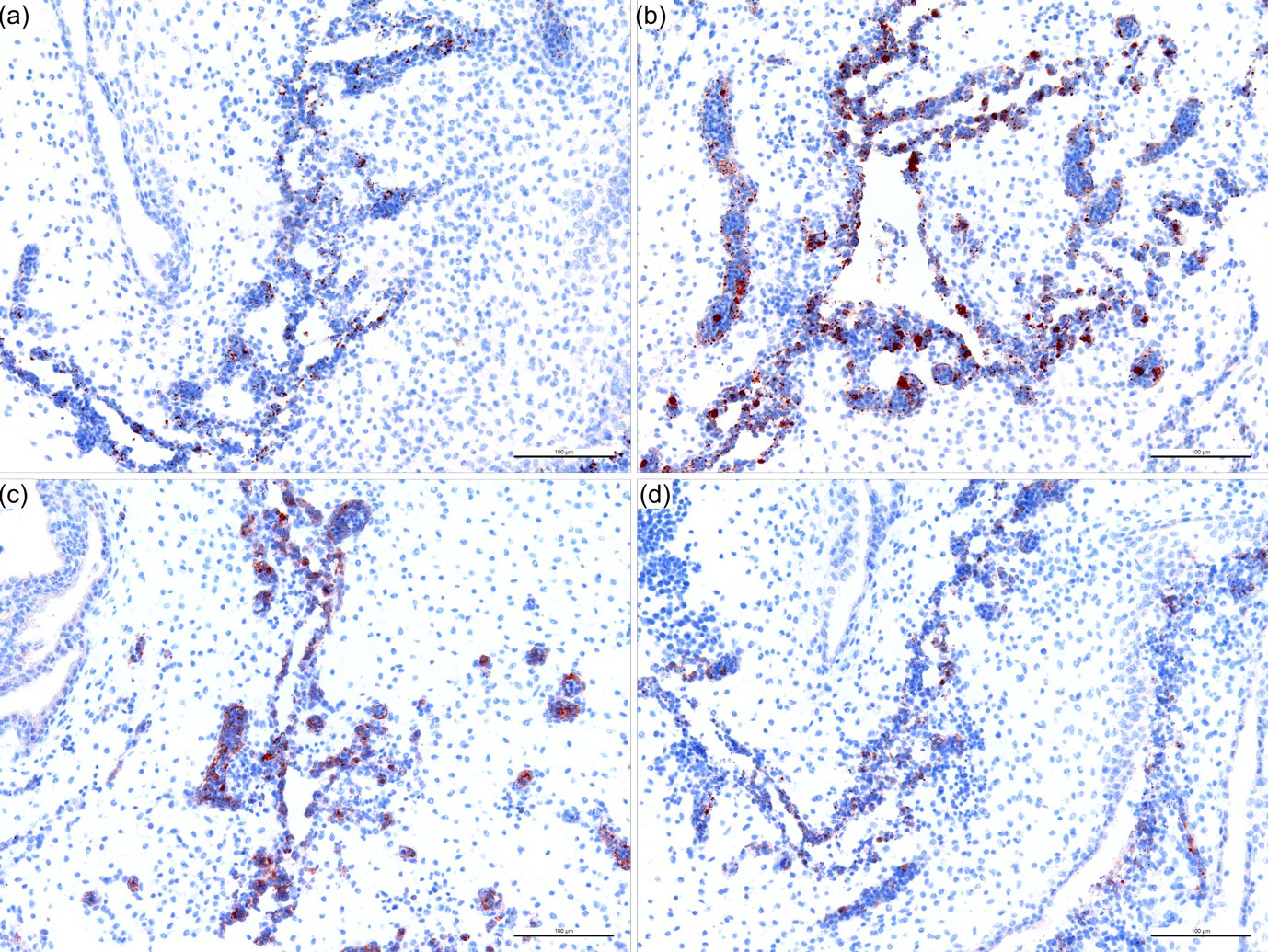
Figure S1. Characteristics of the BTV IHC labelling.** Chorioallatoic membrane immunolabelled against NS1 (a), NS2 (b), NS3/3a (c), VP7 (d). Bar = 100 μm.

**
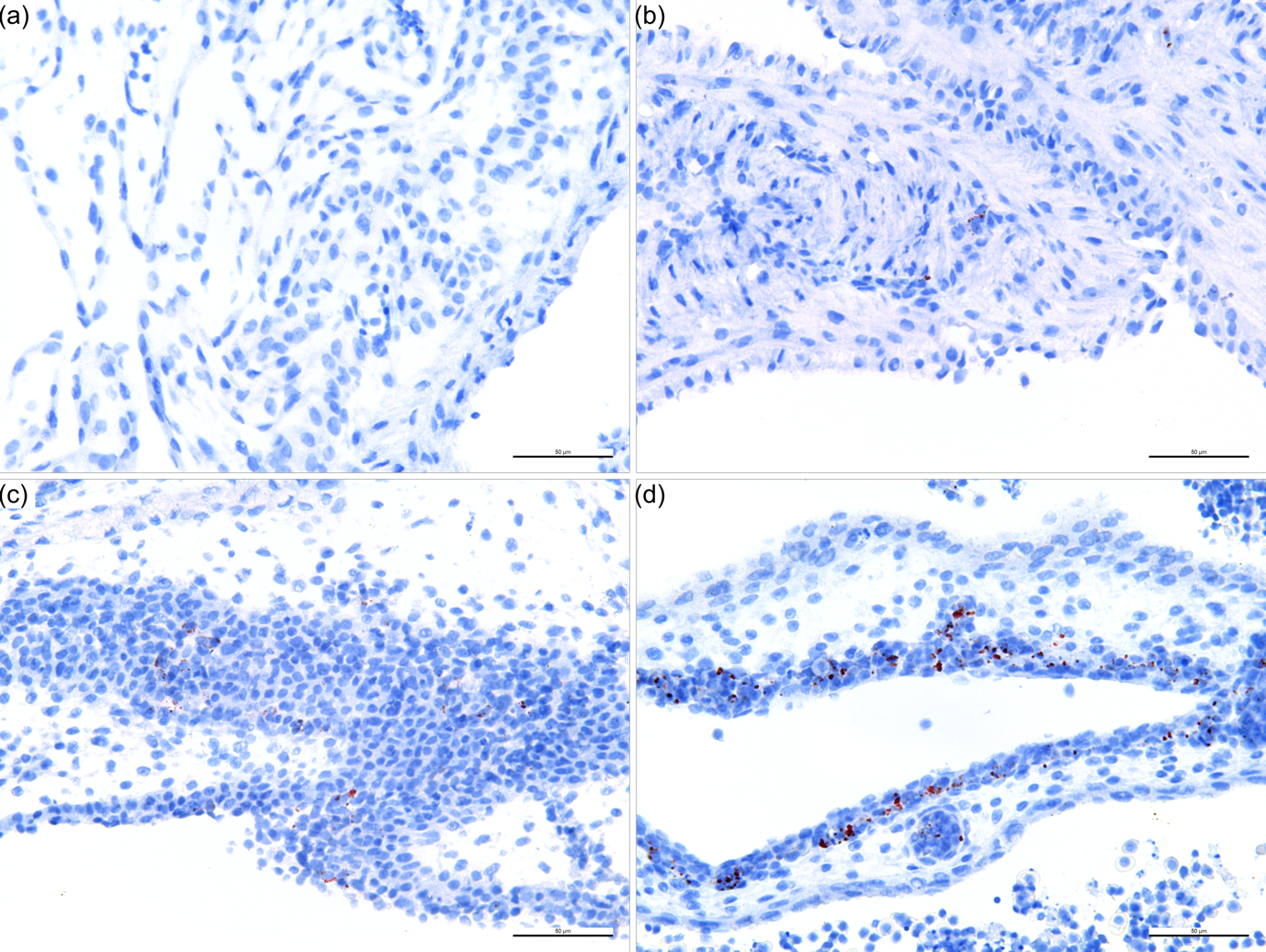
**

**Figure S2. Scoring of BTV IHC.** Chorioallatoic membrane immunolabelled with NS1 antibody. Score=0 (a), score=1 (b), score=2 (c), score=3 (d). Bar = 50 μm.

**Table S1. List of primers used for generation of BTV amplicons**

| **Segment** | **Primer Sequences (5'-3')** | **Genome Positions** |
| --- | --- | --- |
| 2 | AACGCTGTCCCGAGAATGGAG | 7-682 |
|  | TCCTCTGTATTGTATGAAACTG |  |
|  | ACGTGATAGAATTAGCTGAAC | 89-1101 |
|  | AGGGTTGGACCACCACACTCG |  |
|  | ATCGTACCACGCAATCGCTGTT | 462-2100 |
|  | CTTTCGTCGATCGATCGTAGC |  |
|  | AGTGGATGATTCAAGATTCGAT | 335-2100 |
|  | CTTTCGTCGATCGATCGTAGC |  |
|  | GTTCACATGTATGACAGAT | 2041-2935 |
|  | CGTGCAAATGTGAATG |  |
| 5 | AGTTCTCTAGTTGGCAACCA | 9-768 |
|  | ATSGCCATCGAAGCATARCT |  |
|  | GRGTYAGATTGGATGATTC | 431-1260 |
|  | TCTGCTCCAGTGTAACAGT |  |
|  | AGCTGGCTACTACCWATGAT | 950-1751 |
|  | TCTAGTACAGTGCTAAGAGA |  |
| 6 | CATGATCGCGAAAGATG | 15-1493 |
|  | GCATCTGCAATTCATCATCA |  |
|  | AAGATCCCCATGATCGCGA | 7-1636 |
|  | TAAGTGTAAGTCCCGGGATCA |  |
|  | AGATCCCTATGATCGCGAAAGA | 8-689 |
|  | CGATCTCTTGGATGGCTTCCTC |  |
|  | ACGAGATGCGTACGCTAC | 528-1597 |
|  | CTACACGGTGAGAAAGAT |  |
| 7 | AGAGATGGACACTATCGCWGCA | 14-1154 |
|  | AAGTGTAATCTMAGAGACGT |  |
| 8 | AGCARCGTAGATTYACTA | 30-658 |
|  | TCTCGCTTCACGCAGCTTCTCCAA |  |
|  | GGAATTGTGCAACCATATATGC | 395-1057 |
|  | GCTCATGTCYTTAGAGACAA |  |
|  | AGAATGTGCCACTGTACT | 957-1125 |
|  | GTAAGTGTAAAATCCC |  |
| 9 | ATCGCATATGTCAGCTGCGAT | 9-1031 |
|  | TACGCCAAGAAGGTACCCT |  |
| 10 | AAGTGTCGCTGCCATGCTA | 7-793 |
|  | CGTTATACAGCAGTGGGAGT |  |
|  | TGTCGCTGCCATGCTAT | 10-754 |
|  | TCCAAATGCAGCACGTCCCA |  |
|  | TGAGGACAGTAGGTAGAGT | 707-822 |
|  | GCTGTGATGTGTGAATG |  |
